# The Diagnostic Odyssey of Dissociative Identity Disorder: A Case Report of Prolonged Misrecognition

**DOI:** 10.7759/cureus.86278

**Published:** 2025-06-18

**Authors:** Enoch Chi Ngai Lim, Chi Eung Danforn Lim

**Affiliations:** 1 Research and Development, Specialist Medical Services Group, Earlwood, AUS; 2 National Institute of Complementary Medicine (NICM) Health Research Institute, Western Sydney University, Sydney, AUS; 3 School of Life Sciences, University of Technology Sydney, Sydney, AUS

**Keywords:** differential diagnosis, dissociative identity disorder, multidisciplinary care (mdc), multiple personality disorder, psychotherapy in clinical settings

## Abstract

Dissociative identity disorder (DID) is characterised by two or more distinct personality states, often resulting from severe childhood trauma. The disorder is frequently misdiagnosed as depression, anxiety, or borderline personality disorder. This case study is regarding a 28-year-old Caucasian woman with mental health challenges since primary school, including a bullying and domestic violence history, who received multiple incorrect diagnoses (depression, anxiety, panic disorder) given by various psychiatrists, psychologists and general practitioners over several years. Patient treatment with tricyclic antidepressants, selective serotonin reuptake inhibitors, and serotonin-norepinephrine reuptake inhibitors failed to produce any improvements. At age 16, a specialist general practitioner considered the former diagnoses were incorrect and recognised the need for multidisciplinary assessment, leading to referral to a specific psychiatry diagnostic service at a tertiary teaching hospital, where she was correctly diagnosed with DID. Psychologists with a special interest in DID provided the patient with intensive psychotherapy sessions. Her 12-year journey through therapy resulted in significant mental health recovery in addition to finishing her university degree and obtaining a job. This case highlights the importance of recognising DID, challenges in differential diagnosis, and the need for specialised multidisciplinary care.

## Introduction

Dissociative identity disorder (DID) involves the existence of two or more personality states or an enduring personality that is divided into two or more parts, each possessing its own unique mode of thinking and perception [1]. It is placed under dissociative disorders in DSM-5 and involves a disruption in the consciousness, memory, identity, and perception of a person [2]. Previously defined as multiple personality disorder, the term was changed in 1994 to illustrate more accurately the pathology of identity fragmentation [3].

It is estimated that around 1.5% of the world population suffers from DID, and the female-to-male ratio is 1.75-1.9:1 [4]. Patients usually have an average time lapse of 5-12 years between first contact with mental health services and receiving the correct diagnosis [5]. The cause is almost directly associated with severe trauma during childhood, as 95-97% of patients report some form of sexual or physical abuse during childhood [6]. The disorder is associated with psychiatric comorbidities and substantial functional impairment, with 67% of individuals reporting multiple suicide attempts [7]. This case study aims to highlight the importance of recognising DID, challenges in differential diagnosis, and the need for specialised multidisciplinary care.

## Case presentation

A 28-year-old Caucasian woman started to have mental health challenges since her primary school age due to bullying in school as well as domestic violence, specifically a parent physically and verbally abusing her due to behavioural issues, such as frequent emotional outbursts, daydreaming in school, and difficulty maintaining consistent friendships with peers.

Starting from high school at age 12, she consulted various general practitioners and child and adolescent psychiatrists, as well as paediatric psychologists, who, after work-up, diagnosed her with major depressive disorder, generalised anxiety disorder, and panic disorder. She was started on treatments including tricyclic antidepressants, selective serotonin reuptake inhibitors, and serotonin-norepinephrine reuptake inhibitors, which did not yield satisfactory results.

Around the ages of 14 to 16, her symptoms began to intensify in both complexity and severity, moving beyond anxiety and low mood. This period marked a critical shift, as she started experiencing internal fragmentation and dissociative symptoms not previously reported. She noted an astounding contrast between her external presentation and her internal reality in the background. Externally, the patient appeared to be quite cheerful and happy, and somewhat overly energetic, claiming “to pretend to be very friendly” even towards people she did not like. While alone, she described emptiness and “another person living inside her body.”

At age 16, a specialist general practitioner (Specialist GP) considered her presentation to be atypical for depression and anxiety and did not correspond with either expectation in scope. The GP attempted to reconstruct the initial diagnosis and, upon doing so, concluded that the prior diagnoses were likely very far from the mark. Hence, a referral was made to a multidisciplinary psychiatric diagnostic service within a tertiary teaching hospital.

This service encompasses multiple disciplines, including psychiatrists, psychologists, social workers, occupational therapists, and mental health nurses. Following careful considerations, this team identified the coexistence of multiple distinctive personality states, alongside dissociative amnesia, and a range of clinical signs consistent with DID, which turned out to be her first accurate diagnosis after five years spent in the psychiatric system. These included episodes of lost time, sudden shifts in behaviour and demeanour, and the patient’s description of feeling as though “another person” was living inside her body. Observations also noted significant gaps in autobiographical memory and marked inconsistencies in self-representation, further supporting the diagnosis.

After receiving her diagnosis, she had difficulties accessing further treatment due to a shortage of mental health professionals or psychologists with expertise in supporting patients with DID, especially in the public health system. With no other option, the patient could only seek help in the private psychologist market, with the support of her GP. Over a decade, she had to change psychologists three times because her providers relocated or ceased private practice. Nonetheless, she was able to obtain a significant amount of self-understanding, complete her university education, and integrate into the workforce through intensive DID-specific psychotherapy.

## Discussion

Clinical features

DID is characterised by the presence of two or more distinct personality states, accompanied by recurrent gaps in memory and significant disruptions in identity, behaviour, and functioning, not attributable to cultural practices, substances, or medical conditions [8]. The change of states can occur spontaneously or can be triggered, altering voice, posture, and behaviour [9]. Unlike everyday memory lapses, dissociative amnesia affects one's self-related data and recurring activities, distinguishing this type of amnesia from normal forgetfulness [10].

The most notable features are depersonalisation, which involves detachment from the self, and derealisation, a transformation in how the environment is experienced. Additionally, there is dysregulation of emotions, manifested as rapid changes in feelings, and somatic symptoms such as headaches and sleep disturbances [11]. Cognitive symptoms include distractibility and obsessive preoccupation with other identity states within the person [12].

Neurobiological correlation

Emerging neurobiological studies have started explaining the brain mechanisms for DID, and neuroimaging studies have found changes in memory, executive functioning, emotion regulation, and other areas in the hippocampus, amygdala, and prefrontal cortex [13]. These changes indicate that DID is associated with quantifiable neurobiological changes from trauma and dissociation coping mechanisms employed by these individuals.

DID patients' different identity states showed distinct patterns of activation in fMRI studies, supporting the idea that these states represent actual neurobiological changes rather than the mere enactment of roles individuals are playing [14]. In addition, changes in some neurotransmitter systems, especially serotonin, norepinephrine, and gamma-aminobutyric acid, have been documented, which may be responsible for the emotional dysregulation and dissociation characteristic of the disorder [15].

Pitfalls and how to avoid misdiagnosis

Patients with DID usually face a multitude of misdiagnoses, averaging around two to five before receiving the accurate one [5]. Depression tends to take precedence (60-80% of cases) as the most common misdiagnosis, with DID patients displaying mood-related symptoms deemed to be episodic in nature and state-dependent rather than persistent [16].

Anxiety disorders may be confused with DID because the dissociative episodes resemble panic attacks; however, panic disorder is devoid of amnesia and identity confusion, which are essential to a DID episode [17]. Borderline personality disorder (BPD) incorporates both some evidence of emotional dysregulation and identity disturbance. However, while BPD contains an unstable but continuously existing self, DID consists of multiple distinct separate identity states [18].

Psychosis can easily become a misdiagnosis if internal voices are inaccurately diagnosed as hallucinations. In contrast to this, the internal voices associated with DID are distinctive, perpetual characters, and the patient exercises reality testing [19]. Diagnostic strategies include intensive assessment for previous trauma, extended monitoring for changes in identity integration, multidisciplinary review, and identifying patterns of resistance to treatment [20]. Table 1 illustrates the difference between DID and other types of personality disorders. 

**Table 1:** Differential Diagnosis: Dissociative Identity Disorder vs. Personality Disorders vs. Borderline Personality Disorder Original Table created by authors

Feature	Dissociative Identity Disorder (DID)	Personality Disorders (PD)	Borderline Personality Disorder (BPD)
Core Pathology	Identity/memory disruption from trauma	Maladaptive personality traits	Emotional dysregulation
Identity Disturbance	Distinct separate identity states	Consistent maladaptive traits	Unstable self-image with continuity
Memory Function	Extensive dissociative amnesia	Generally intact	Occasional stress-related gaps
Trauma History	95-97% severe childhood trauma	Variable	80-96% childhood trauma
Consciousness	Altered states, switching episodes	Normal consciousness	Normal with brief dissociative episodes
Treatment Response	Poor to conventional medications	Variable	Moderate to Dialectical Behaviour Therapy (DBT) or specialised treatments

DID is characterised by separate, distinct identity states with their own memories, unlike BPD's Identity Diffusion, which maintains some form of continuity [21]. Altered memories in DID are more structurally comprehensive and meticulous [22]. Both conditions may co-occur in 25-50% of the cases [23].

From the perspective of general practitioners and other frontline health practitioners, including psychologists and psychiatrists, avoiding misdiagnosis of DID requires an informed, trauma-aware approach. It is important to differentiate internal voices from auditory hallucinations typically seen in psychotic disorders; in DID, these voices are experienced as internal, distinct, and often conflicting entities, but with preserved reality testing. Clinicians should remain vigilant for red flags such as identity disruption, frequent memory gaps, shifts in mood or behaviour, and inconsistencies in the patient's reported experiences. A detailed psychosocial history, including past trauma and adverse childhood experiences, is crucial. Use of screening tools like the Dissociative Experiences Scale (DES), along with early referral to trauma-informed mental health specialists, can facilitate accurate diagnosis and reduce the risk of inappropriate labelling and treatment.

DID often first presents during a child's school years, placing teachers in a vital frontline position for noticing signs and providing support. Educators may observe erratic behaviour, frequent daydreaming or blank moments, sudden shifts in mood or voice, memory gaps that bewilder the child, and difficulties in maintaining friendships. Instead of hastily labelling the student as problematic, teachers can respond with empathy and a trauma-informed perspective that views the root as past harm. Creating a calm, predictable classroom and regularly communicating with the school counsellor helps establish a safety net. If concerning signs persist, teachers should collaborate with the wellbeing team or directly refer the child to the on-site psychologist. Early discussions with parents, and, where appropriate, recommendations for further assessment by a child and adolescent mental health service or the family's general practitioners, can ensure timely diagnosis and care, which may spare everyone years of mislabeling and distress. 

Management of DID

**Figure 1:**
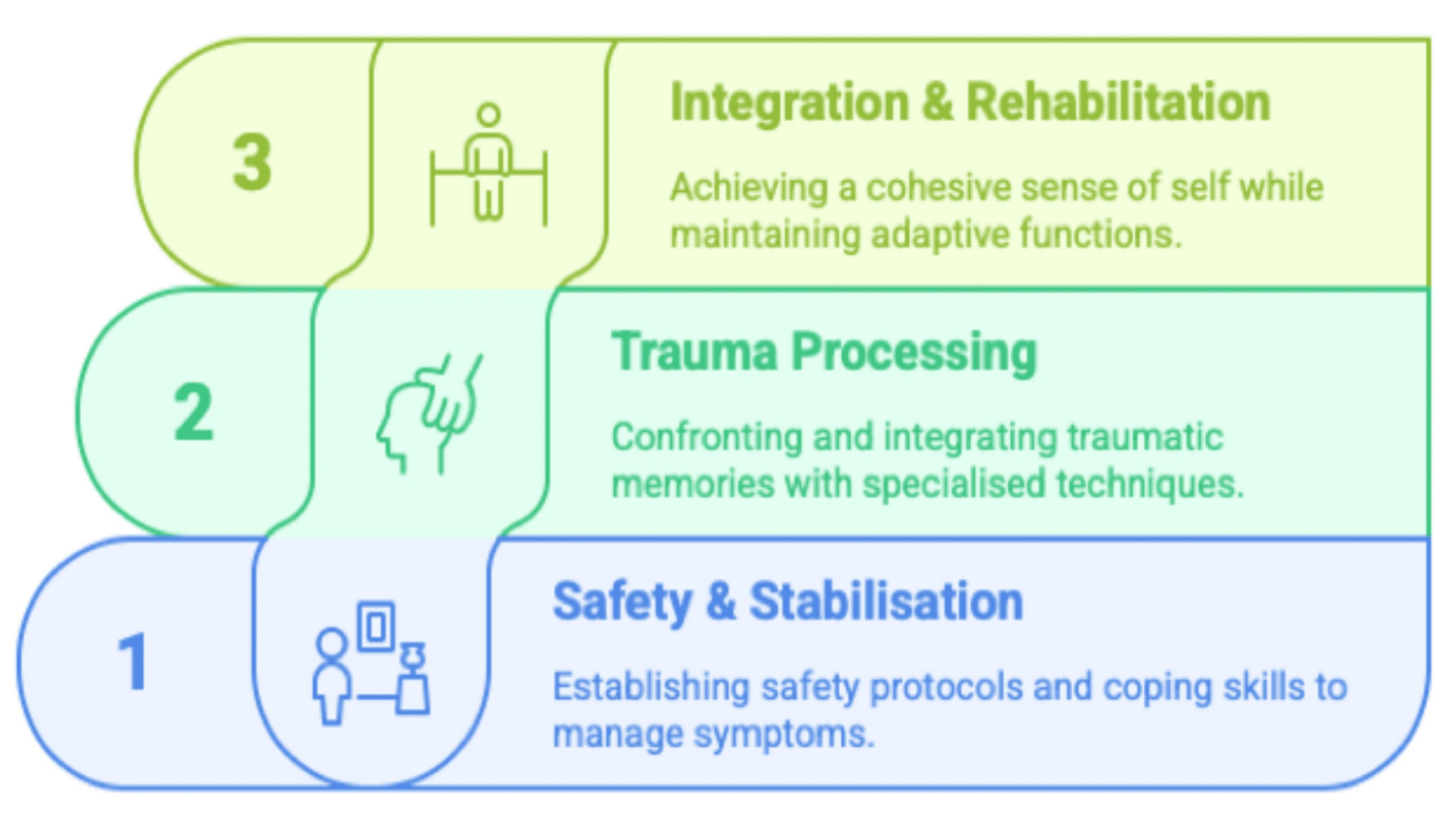
Three-Phase Management of DID Original figure created by the authors DID: Dissociative identity disorder

DID requires specialised phase-based treatment according to the International Society for Study of Trauma and Dissociation guidelines [24]. These are divided into three stages: safety and stabilisation, trauma processing, and integration and rehabilitation [25], as illustrated in Figure 1.

In phase 1, the focus is on developing a comprehensive safety plan, which is crucial given the history of suicide attempts, psychoeducation and coping skill enhancement. These are critical first steps, as two-thirds of the population has a suicide attempt history (67%) [7]. Step two is cautious trauma processing that considers multiple identity states (phase 2) [26]. The third phase focuses on integrating functional identity, secondary role identity, and multifaceted rehabilitation [27].

DID adapted eye movement desensitisation and reprocessing therapy, trauma-focused cognitive behavioural therapy, or psychodynamic psychotherapy may be used [28]. There are no specific medications for DID due to its unique traits; instead, pharmacotherapy aims to alleviate accompanying symptoms [29]. When tailored care is received, results are generally positive, albeit long-term treatment is required [17].

Take home messages

Recognition of DID during its early stages is fundamental due to the average 5-12-year delay with multiple incorrect diagnoses [5]. Given the strong trauma-DID association, comprehensive trauma assessment is warranted [30] as part of the mental health review during consultation. Diagnostic precision improves with multidisciplinary assessment of complex presentations [31]. Specialised treatment is needed, highlighting the need for qualified clinician training [32]. As shown in this case, where the patient achieved functional recovery, positive outcomes are possible when proper, enduring care is given [33].

## Conclusions

This case report illustrates the dual diagnostic and care dilemmas that accompany DID, while highlighting the attainable positive outcomes with adequate treatment. The years of being misdiagnosed to recovery embody the complexity of DID and the importance of receiving specialised care. This case emphasises the need for better acknowledgement of DID, including comprehensive trauma assessment, multidisciplinary approaches, and a shift in focus for healthcare systems to train clinicians for specialised, enduring treatment of complex trauma-related disorders.
